# The Successful Anesthetic Management of a Cesarean Delivery in a Patient with Fanconi–Bickel Syndrome

**DOI:** 10.1155/2022/3220486

**Published:** 2022-07-08

**Authors:** Alexander M. DeLeon, Rishi D. Gaiha, Feyce M. Peralta

**Affiliations:** ^1^Northwestern Memorial Hospital, 251 East Huron Suite F5-704, Chicago, Illinois 60611, USA; ^2^Advocate Illinois Masonic Hospital, 836 West Wellington Avenue, Chicago, Illinois 60657, USA

## Abstract

**Introduction:**

Fanconi–Bickel syndrome (FBS) is a rare genetic condition characterized by extremely short stature, renal tubular dysfunction, osteoporosis, and rickets. The literature is scant regarding the successful reproduction of women with FBS. Cesarean delivery is indicated due to the risk of pelvic fracture from vaginal delivery in a patient with FBS and rickets, yet no case describing the anesthetic has been reported. *Clinical Findings*. We present a 39-inch-tall, 46.47 kg/m^2^ BMI woman with FBS who was scheduled for cesarean delivery and requesting neuraxial anesthesia. A low-dose, combined spinal-epidural technique (CSE) was employed to account for her extreme short stature yet allow for additional dosing if needed. The cesarean delivery, hospital course, and follow-up were all uneventful.

**Conclusion:**

A single case of an FBS patient's successful pregnancy was previously reported in the literature without describing the anesthetic technique. Our case is unique in that it is the first case in the literature that we are aware of describing the anesthetic technique. This case may provide a template for clinicians treating FBS patients and patients with extremely short stature.

## 1. Introduction

Fanconi–Bickel syndrome (FBS) is a rare autosomal recessive condition characterized by hepatorenal glycogen accumulation, extremely short stature, hypo- and hyperglycemia, and proximal renal tubular dysfunction [1]. FBS results from a defective monosaccharide transporter, Glut2, in cell membranes [2]. Rickets and osteoporosis are later life findings associated with FBS and pathological fractures [1]. Eighty-two cases from 70 families were reviewed in 1998, and we know only a single report of reproduction in a person with FBS in the literature [1, 3].

We report a case of a pregnant patient with FBS presenting for primary cesarean delivery requesting neuraxial anesthesia. FBS patients with rickets are presumed to be at risk for pelvic fracture from vaginal delivery, and thus cesarean delivery is indicated. To our knowledge, a case has not been published describing the anesthetic technique for successful delivery in a patient with FBS [3]. Our patient's short stature (height 39 inches) and high BMI (46.47 kg/m^2^) presented the authors with a clinical challenge when developing an anesthetic plan. This report aims to describe the rationale behind our anesthetic choices which contributed to a successful outcome. The principles can apply to FBS patients and other patients presenting for cesarean delivery with extremely short stature. The authors obtained permission and written informed consent from the patient presented in this case. The CARE case report guidelines and checklist were followed in the preparation of this paper [4].

## 2. Case Description

A 22-yo-G2P0 woman at 37-week gestation presented with a history of Fanconi–Bickel syndrome, short stature, rickets, and congenital aortic root dilation. She was followed by cardiology with serial echocardiography demonstrating a stable aortic root. The patient was also followed by nephrology to stabilize electrolyte levels. Her medications included calcitriol, calcium, potassium-phosphate, potassium-chloride, and insulin lispro.

The patient's pregnancy was notable for type A2 gestational diabetes mellitus (A2GDM). She also required supplemental tube feeds, which she received through a percutaneous gastric tube (G-tube). She experienced several episodes of hypoglycemia, requiring adjustments to her insulin dosing and tube feeds. The obstetrician advised the patient that a cesarean delivery would be optimal given her history of rickets and her increased risk for pelvic fractures.

**Figure 1:**
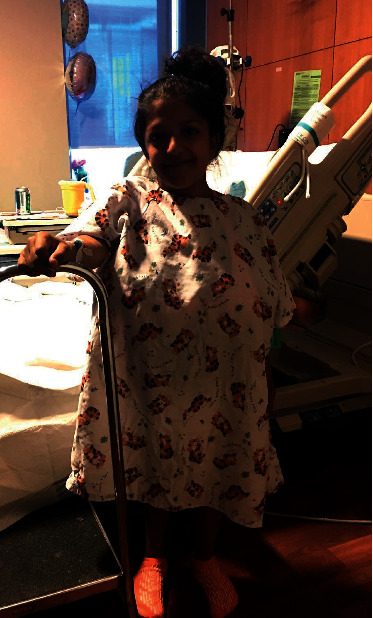
Fanconi–Bickel syndrome patients demonstrate characteristic short stature.

**Figure 2:**
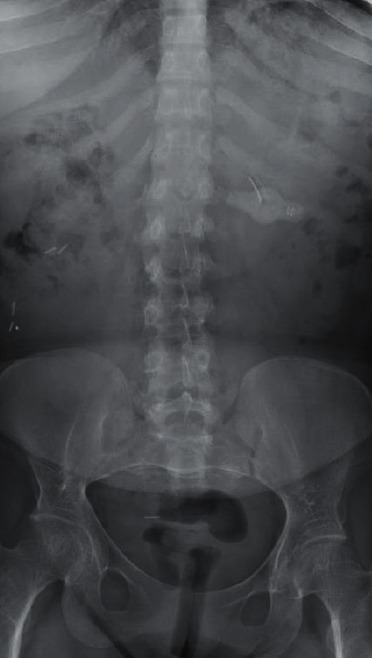
The patient demonstrated normal spine anatomy.

The patient's preoperative vital signs were BP 103/67 mmHg, HR 84 bpm, RR 16/min, weight 45.6 kg, height 39 inches, and BMI 46.47 kg/m^2^. Her physical exam revealed extremely short stature (see Figure 1), normal heart and lung exam, Mallampati class I, full range of motion in the neck, and a normal lumbar spine exam. Her calcium was low at 8.2 mg/dL (reference range 8.3–10.5 mg/dL). Her alkaline phosphatase was elevated (259 IU/L, reference range 34–104 units/L), and her total protein was low at 6.0 g/dL (reference range 6.4–8.9 g/dL). All other chemistries, complete blood count, liver function tests, and coagulation studies were within normal ranges. A pre-pregnancy abdominal X-ray demonstrated normal spine anatomy with degenerative changes (Figure 2).

Before arriving in the operating suite, the patient was premedicated with 50 mg of ranitidine, 10 mg of metoclopramide, and 30 mL of sodium citrate. Her anesthetic plan consisted of a combined spinal-epidural technique (CSE) with 7.5 mg of intrathecal hyperbaric bupivacaine and 10 mcg of intrathecal fentanyl. Intrathecal morphine was not administered. A phenylephrine infusion was used to maintain adequate maternal blood pressure. After her combined spinal anesthetic administration at the L3-4 level on the second attempt, the catheter was secured at 10 cm at the skin with a recorded loss of resistance to air at 5.0 cm. Three minutes after intrathecal dosing, the patient's BP decreased from 133/72 mmHg to 111/60 mmHg, and her HR dropped from 94 bpm to 61 bpm. No additional vasopressors were required to treat the hemodynamic changes, and the patient remained asymptomatic.

The cesarean delivery proceeded uneventfully with 700 mL of estimated blood loss, 1800 mL of crystalloid administered, 17.4 units of oxytocin given, and a final measured sensory level of T1 bilaterally 55 minutes after initial CSE placement. The epidural catheter was never used, nor was a test dose administered, and the epidural catheter was removed before transfer to the postoperative recovery room. Discharge criteria were met 100 minutes after arrival in the recovery room, and the remainder of the patient's hospital course was uneventful.

## 3. Discussion

Our patient's short stature and BMI of 46.47 kg/m^2^ presented a clinical challenge when planning a neuraxial anesthetic. Given the patient's normal airway exam, a general anesthetic would have been an appropriate choice. Hawkins et al. demonstrated that neuraxial anesthesia provided no statistically significant mortality benefit compared to general anesthesia [5]. Despite the questionable mortality benefit, other benefits to neuraxial anesthesia exist, including maternal bonding, the possibility of skin-to-skin contact, reduced blood loss, beneficial postoperative pain profile, and possibly lower thrombotic risks [6]. Given the patient's normal spine anatomy, patient preference alone was the primary driver for pursuing neuraxial anesthesia in this patient without other contraindications. The decision to pursue a CSE vs. traditional epidural was based on the improved outcomes associated with spinal anesthetics for elective cesarean deliveries [7].

Wei et al. published a study in 2017 indicating variables that affect the spread of intrathecal hyperbaric bupivacaine in pregnant patients [8]. The authors showed that more considerable abdominal girth and shorter vertebral column length were predictive of a higher cephalad spread of local anesthetic. The authors found the correlation coefficient between high abdominal girth and spinal anesthetic spread to be 0.372. Wei et al. also found the correlation coefficient between vertebral column length and spinal spread to be −0.711 [8]. Those findings suggest decreasing our usual dose of hyperbaric bupivacaine for our patient's large abdominal girth and her short vertebral column length. Our standard dose of local anesthetic for an elective cesarean delivery is 12.0 mg of hyperbaric bupivacaine. We decided to administer 7.5 mg of hyperbaric bupivacaine to balance the risk of a high spinal block with the risk of failed spinal anesthetic. An epidural catheter was placed as part of the CSE technique to be utilized if a surgical level could not be obtained from the spinal block or if the surgery outlasted the duration of the intrathecal dose. Our patient demonstrated a spinal level adequate for surgery without excessive hemodynamic changes or prolonged recovery.

Neuraxial morphine is considered the standard of care for postoperative analgesia for patients undergoing cesarean delivery. The Society for Obstetric Anesthesia and Perinatology published a consensus statement addressing monitoring recommendations for preventing and detecting respiratory depression associated with administering neuraxial morphine for cesarean delivery analgesia [9]. Although they list a set of risk factors for respiratory depression, the risk of respiratory depression in patients with extremely short stature remains unknown. Therefore, the authors opted to avoid administering intrathecal morphine for this case.

We report this interesting case to give a template for future clinicians dealing with FBS patients undergoing cesarean delivery. The greatest strength of this report is that it is possibly the first case of successful neuraxial anesthesia for cesarean delivery in an FBS patient. Yet, the limitations of this report must be considered. We offer a single patient, yet given that each patient will have unique anatomy, it is difficult to apply our plan to all FBS patients universally. The specific height, weight, spine anatomy, and BMI should be considered when treating any patient with extremely short stature. Also, our patient requested neuraxial anesthesia, yet general anesthesia is an acceptable choice given no other contraindications.

The literature regarding FBS in pregnancy is scant. A single case published in 2011 describes a successful cesarean delivery in a patient with FBS [3]. No description of the anesthetic technique was provided in that report. To our knowledge, this case report represents the first description of a successful neuraxial anesthetic in a pregnant patient with Fanconi–Bickel syndrome presenting for cesarean delivery. We recommend our CSE technique with a low initial spinal dose (e.g., 7.5 mg of hyperbaric bupivacaine). We believe that the spinal dose provides surgical anesthesia, and the placement of an epidural catheter allows for the extension of the spinal level if necessary. We offer this case to guide future clinicians treating FBS patients, which also may be applied to any pregnant patient with extremely short stature.

## Data Availability

The data used to support this case report are included within the article.
